# Altered lipid peroxidation, perineuronal net and oligodendrocyte markers in the frontal cortex of a dual-hit neurodevelopmental model support its relevance to schizophrenia

**DOI:** 10.3389/fncel.2026.1784522

**Published:** 2026-03-16

**Authors:** Victoria Esuaikoh, Sarah Ibegbulam, Alistair Cook, Bianca McFarland, Nora Nunnington, Ambalangoda Perera, Jingjing Feng, Jennifer A. Cale, Madeleine V. King

**Affiliations:** MIND Laboratory for Preclinical Research into Mental Illness and Neurodivergence, Division of Physiology Pharmacology and Neuroscience, School of Life Sciences, University of Nottingham, Nottingham, United Kingdom

**Keywords:** frontal cortex, isolation rearing, neonatal PCP, oligodendrocyte, oxidative stress, perineuronal net, schizophrenia, synaptic density

## Abstract

**Introduction:**

The pathogenesis of schizophrenia begins in early neurodevelopment and leads to an array of frontal cortical deficits. They include redox dysregulation, white matter perturbation, loss of perineuronal nets (PNNs) and reduced synaptic density. It is therefore highly desirable that preclinical models used to understand disease, select drug targets and evaluate novel therapeutics encompass similar changes. One approach to improved preclinical modeling incorporates dual-hit neurodevelopmental interventions, like neonatal administration of phencyclidine (PCP, to disrupt development of glutamatergic circuitry) then post-weaning isolation (Iso, to mimic adolescent social stress). We recently showed that rats exposed to PCP-Iso develop GABAergic and inflammatory changes in the frontal cortex, and the current study expands on this by comparing changes to additional cellular and extracellular matrix markers relevant to oxidative stress, myelination, PNN integrity and synaptic vesicle density.

**Methods:**

The study used tissue from a previously described cohort of male Lister-hooded rats. They received saline vehicle (Veh, 1 ml/kg s.c.) or PCP (10 mg/kg s.c) on postnatal days (PND) 7, 9 and 11 then were housed in social groups (Gr, 3–4/cage) or post-weaning isolation from PND 21 onwards. Declarative memory was assessed in adulthood (PND 57–80) using a novel object discrimination (NOD) test. Frontal cortical samples were obtained on PND 79–80 and used for immunohistochemical or lectin binding examinations of the lipid peroxidation product 4-hydroxynonenal (4-HNE), oligodendrocyte-associated protein 2′,3′-cyclic nucleotide 3′-phosphodiesterase (CNPase), PNNs and synaptic vesicle glycoprotein 2A (SV2A) throughout the orbitofrontal, prelimbic and infralimbic cortices. In each case data from dual-hit PCP-Iso and single-hit Veh-Iso were compared to each other, and to data from Veh-Gr controls.

**Results:**

Single-hit isolation-reared and dual-hit PCP-Iso both showed impaired declarative memory. They also both exhibited reduced PNN density in the orbitofrontal cortex and reduced PNN thickness in the prelimbic/infralimbic cortex. However, PCP-Iso showed additional PNN thinning, 4-HNE upregulation and CNPase downregulation in orbitofrontal regions.

**Discussion:**

These findings enhance the face validity of PCP-Iso and support wider use of this preclinical model for evaluating novel therapeutics designed to support parvalbumin-positive neurons and PNNs, promote myelination or normalize redox dysregulation. Unaltered SV2A expression in young adult PCP-Iso mirrors recent dorsolateral prefrontal cortical findings in first-episode psychosis, supports expectations that increased microglial activation precedes aberrant synaptic pruning, and justifies further examinations of synaptic markers in PCP-Iso at later developmental stages.

## 1 Introduction

Schizophrenia is a complex disorder that ranks among the top 10% causes of disability worldwide (Velligan and Rao, 2023). The pathogenesis begins in early neurodevelopment and is believed to include aberrant microglial-mediated synaptic pruning and cortical excitatory-inhibitory imbalance (Howes and Onwordi, 2023). For example, immunohistochemical studies show increased microglial activation within the frontal cortex, including higher densities of cells expressing the major histocompatibility complex class II antigen HLA-DR (Radewicz et al., 2000; Wierzba-Bobrowicz et al., 2005; Fillman et al., 2013), as well as a shift toward a more activated amoeboid morphology of cells labeled for ionized calcium binding adaptor molecule 1 (Iba-1; Hercher et al., 2014). Imaging studies demonstrate lower gray matter volumes and reduced density of a synaptic terminal marker, synaptic vesicle glycoprotein 2A (SV2A; Howes et al., 2023). There is also post-mortem evidence for lower levels of parvalbumin mRNA (Mellios et al., 2009; Hoftman et al., 2015; Chung et al., 2016), and fewer parvalbumin-immunoreactive neurons (Beasley and Reynolds, 1997; Glausier et al., 2014) or reduced intensity of parvalbumin immunostaining (Enwright et al., 2016). Redox dysregulation has been proposed as one of the initial triggers (Perkins et al., 2020). In addition to stimulating microglial activation it leads to white matter deficits and degradation of the protective perineuronal nets (PNNs) that predominantly ensheath parvalbumin-positive interneurons. Consistent with this, post-mortem frontal cortical samples from patients with schizophrenia show increased expression of the lipid peroxidation product 4-hydroxynonenal (4-HNE; Wang et al., 2009; Andreazza et al., 2013), reduced expression of the oligodendrocyte-associated protein and myelination indicator 2′,3′-cyclic nucleotide 3′-phosphodiesterase (CNPase; Flynn et al., 2003; Wesseling et al., 2014), and reduced expression of PNN markers (Lisboa et al., 2024). These changes all represent potential targets for novel disease-modifying therapeutics, so it is highly desirable that they feature in the models used for preclinical evaluation. This is a realistic expectation for models based on genetic (Cardis et al., 2018) and/or neurodevelopmental approaches (Lodge et al., 2009) but cannot be achieved with acute pharmacological impairments.

The current study involved lectin-binding and immunohistochemical assessments of PNNs, 4-HNE, CNPase and SV2A in samples from a dual-hit neurodevelopmental model that our group have established and characterized over the past 10 years. It is based on the combination of two established approaches: administration of the NMDA receptor antagonist phencyclidine (PCP) during the neonatal period, followed by post-weaning isolation rearing (Iso) of gregarious rat pups in individual cages to mimic social isolation throughout adolescence. Although there are current and historical reports of infants being exposed to PCP at the equivalent stage in prefrontal cortical development (around birth; Chini and Hanganu-Opatz, 2021) due to recreational use by mothers (Mvula et al., 1999; Habersham et al., 2025) these individuals have either not undergone systemic follow up for psychiatric illness or not yet reached an age where this would be appropriate. We therefore do not wish to imply a causal link to recreational PCP exposure in most cases. Rather, these interventions were selected because short-term blockade of NMDA receptors in early life triggers widespread apoptotic neurodegeneration in the developing brain, including an 8.5- to 22.5-fold increase in frontal cortical apoptosis at the dose and ages used here (Ikonomidou et al., 1999). Furthermore, the latest meta-analysis shows that adversity (particularly physical and emotional neglect) before the age of 18 is associated with a 3.3-fold increase in the risk of psychosis and an earlier onset of symptoms (Zhou et al., 2025). The PCP-Iso combination induces a more robust behavioral phenotype than either manipulation alone, spanning negative valence, cognitive and social components of the Research Domain Criteria (RDoC; Gaskin et al., 2011, 2014; Kohli, 2018; Watson et al., 2016). These are accompanied by GABAergic, glutamatergic and dopaminergic changes in the hippocampus (Gaskin et al., 2016; Shortall et al., 2020) as well as reduced density of parvalbumin-positive interneurons, increased IL-6 levels and increased microglial activation within the frontal cortex (Cale et al., 2024). The current work builds on these findings through novel assessments of PNNs and markers of lipid peroxidation, mature oligodendrocytes and myelination, and synaptic vesicle density. Findings provide an important backdrop against which to interpret neurochemical substrates of the accompanying declarative memory deficits (Shortall et al., 2020) in the same animals. Replication of changes reported in patient groups (Flynn et al., 2003; Wang et al., 2009; Andreazza et al., 2013; Wesseling et al., 2014; Lisboa et al., 2024) would support wider use of the PCP-Iso model for assessment of new therapeutics.

## 2 Materials and methods

### 2.1 Animals and experimental design

This research used stored brain tissue from a previous cohort of 42 male Lister hooded rats. They have been extensively described in our previous publications, where PCP-Iso showed impaired declarative memory in a novel object discrimination (NOD) test and reduced hippocampal calbindin expression (Shortall et al., 2020), together with reduced parvalbumin immunoreactivity, increased microglial activation and increased IL-6 levels in the frontal cortex (Cale et al., 2024). All procedures were conducted in accordance with the Animals (Scientific Procedures) Act, 1986, with approval from the University of Nottingham Animal Welfare and Ethical Review Body (AWERB). The work was designed and is reported in accordance with the Animal Research: Reporting of *In Vivo* Experiments (ARRIVE) guidelines (Percie du Sert et al., 2020).

In summary, rats from a total of 6 l were obtained with dams on postnatal day (PND) 3 (Charles River UK). They were maintained under controlled conditions throughout the study (21 ± 2 °C, 55 ± 10% humidity, 12-h light-dark cycle; on at 07:00 h) with unlimited access to food (2918 Teklad Irradiated Global 18% Protein Rodent Diet; Innotiv) and water (in polycarbonate bottles with stainless steel sipper tubes; Tecniplast). Upon arrival, family groups were housed in individually ventilated cages (GR1800 Double-Decker; Tecniplast) containing sawdust bedding and standard environmental enrichment (cardboard play tube, wooden chew block and paper nest material). One pup died between delivery and the start of the study and the remaining 41 pups, who each represented a single experimental unit, were randomized (by drawing lots) to receive neonatal administration of sterile saline vehicle (Veh, DEMO S.A.; 1 mL/kg s.c.) or PCP HCl (10 mg/kg base; Sigma-Aldrich) on PND 7, 9 and 11. This ensured each family group included a mix of both vehicle- and PCP-treated pups, which is an important design consideration to avoid any chance of litter effects confounding the resulting data. Although it introduces the possibility that dams could ingest PCP during licking and grooming of drug-treated offspring and passing it to suckling control offspring via the milk, the dose “vehicle-treated” pups might experience through this route represents approximately 2% of that encountered by PCP-treated pups and the long-term consequences of this are likely to be minimal (Chakrabarti and Law, 1983; Cale et al., 2024).

At weaning age (PND 21) the Veh-treated rats were further randomized (again by drawing lots) to rearing in standard groups of three or four per cage (Veh-Gr control; *n* = 14) or isolation i.e., one per cage (single-hit Veh-Iso; *n* = 13). PCP-treated rats were all isolated (PCP-Iso dual hit; *n* = 14). The cohort did not include single-hit PCP-Gr animals because we have already shown they do not exhibit cognitive dysfunction or lasting excitatory-inhibitory imbalance (Gaskin et al., 2014; Shortall et al., 2020). The focus of our current immunohistochemistry was to further understand the differences between Veh-Iso and PCP-Iso rather than their absence in PCP-Gr, and the present approach allowed us to reduce total animal use by 25%. Group sizes were based on previous studies employing the same techniques (Gilabert-Juan et al., 2013; Gaskin et al., 2014; Kohli, 2018). Following weaning, rats were housed in cages (Gr: 32 × 51 cm, Iso: 25 × 42 cm) containing sawdust bedding without environmental enrichment, and which had grid lids to ensure maintenance of visual, olfactory, and auditory contact between isolation-reared rats and other group- and singly-housed rats within the same holding room (Fone and Porkess, 2008). Handling was restricted to a single weekly cage change and body weight measurement until behavioral testing.

To assess differences in pharmacological reversal of declarative memory deficits between Veh-Iso and PCP-Iso, rats underwent NOD (as described in detail elsewhere; Shortall et al., 2020) on three separate occasions at 1-2 week intervals (PND 57-80). They received a single i.p. administration of 0.5% methylcellulose 1% Tween-80 vehicle (1 mL/kg), the 5-HT_6_ receptor antagonist SB-399885 (10 mg/kg; Sigma-Aldrich) or mGlu_7_ antagonist MMPIP (10 mg/kg; Tocris), on separate test days, using a cross-over design and in a pseudorandom order. It would be highly unlikely for these acute treatments to impact on the immunohistochemical data presented here or in our prior manuscripts from the same study (Shortall et al., 2020; Cale et al., 2024), and treatment order was fully balanced across Veh-Gr, Veh-Iso, and PCP-Iso groups to avoid any potential confounds based on recency. The humane endpoint would have been euthanasia of any rat experiencing a decrease in body weight (up to a maximum permitted limit of −20%) and/or signs of poor body condition (e.g., piloerection, hunched posture, absence of grooming) although in practice none of these were encountered. Rats were killed by concussion and immediate decapitation on PND 79–80, straight after the final NOD test. This method was selected to avoid the recognized aversive effects of CO_2_ exposure (Conlee et al., 2005) and to enable rapid extraction and freezing of brain regional samples, which was important for previous neurotransmitter and cytokine analyses involving the opposite hemispheres of the same rats (Cale et al., 2024). For immunohistochemistry the remaining intact hemispheres were immerse fixed in 4% paraformaldehyde (overnight at 4 °C), cryopreserved in 0.1 M Phosphate Buffered Saline (PBS; Oxoid) containing 30% sucrose (Scientific Laboratory Supplies; also overnight at 4 °C), then frozen in isopentane (Fisher Scientific) on dry ice and stored at −80 °C. Full blinding of experimenters to neurodevelopmental history throughout the 8–9 weeks of post-weaning housing was not possible due to the obvious visual difference between group and single housing. However these allocations were concealed throughout tissue processing and analysis.

### 2.2 Lectin-binding and immunohistochemistry

Serial coronal sections (60 μm) were obtained throughout the frontal cortex (Bregma 5.20 to 4.00; Paxinos and Watson, 2006) using a freezing microtome (Anglia Scientific). They were stored in antifreeze solution containing 30% ethylene glycol (Fisher Scientific) and 30% glycerol (Honeywell) in PBS at −20 °C until free-floating immunohistochemistry, which began with washes (4 × 5 min) in PBS to remove antifreeze. Tissue from one Veh-Gr and one Veh-Iso was excluded due to technical difficulties during slicing, and that from two further Veh-Gr and one PCP-Iso was exhausted during previous measures (Cale et al., 2024). This resulted in final group sizes of *n* = 11 Veh-Gr, *n* = 12 Veh-Iso, and *n* = 13 PCP-Iso here. These are sufficient for the planned histochemical examinations, despite being lower than those needed for behavioral work. An average of four evenly spaced sections per animal were processed for each of the four selected markers.

For 3,3′-diaminobenzidine (DAB) labeling of PNNs sections were incubated (30 min) in a solution of 1.5% H_2_O_2_ (Sigma-Aldrich), 10% methanol (Honeywell) and 0.4% Triton-X100 (Sigma-Aldrich) in PBS, to block endogenous peroxidase activity, then washed (2 × 5 min) with PBS. They were incubated (1 h) in 5% normal horse serum (Vector Laboratories) and 0.4% Triton-X100 in PBS to minimize non-specific binding, followed by (overnight, 4 °C) Wisteria floribunda agglutinin (WFA; Vector Laboratories, B-1355-2, 1:1000 in blocking solution) which is a lectin that binds chondroitin sulfate chains in PNNs. Sections were washed (3 × 5 min) in PBS, incubated (45 min) in Vectastain^®^ avidin-biotin complex (ABC solution that had been pre-mixed for 30 min; Vector Laboratories, PK-4000), washed (2 × 5 min) in PBS then incubated (45 s) in DAB solution with nickel (Vector Laboratories, SK-4100). After final washes (3 × 5 min) in dH_2_O, sections were mounted on gelatinized slides and air-dried. Tissue was dehydrated through increasing concentrations of ethanol (70% in dH_2_O, 90% in dH_2_O, 100%; 5min each) then slides were washed (2 × 5 min) with HistoClear II (Scientific Laboratory Supplies), allowed to dry completely and cover slipped with DPX mounting medium (Merck).

For fluorescent labeling of 4-HNE, CNPase and SV2A sections were incubated (1 h) in 2% normal goat serum (Abcam) plus 2% normal donkey serum (Sigma-Aldrich) in buffer 1 (0.5% bovine serum albumin (BSA; Sigma-Aldrich) and 0.3% Triton-X100 in PBS) to minimize non-specific binding of secondary antibodies to the tissue. Sections were incubated (overnight, 4 °C) in rabbit polyclonal antibody against CNPase (Abcam ab227218, 1:400), or recombinant rabbit monoclonal antibody against SV2A (Abcam ab254351, 1:500) plus mouse monoclonal antibody against 4-HNE (Abcam ab48506, 1:275), then washed (3 × 5min) in buffer 2 (0.15% BSA and 0.1% Triton-X100 in PBS) to prevent any unbound primary antibody from interacting with goat anti-rabbit Alexa-Fluor 568 and donkey anti-mouse Alexa-Fluor 488 secondary antibodies (Abcam ab175471 and ab150105, 1:500; 1 h in the dark). The manufacturers technical information stated that the selected primary antibodies produced bands of the expected molecular weights during western blotting (CNPase 48kDa, SV2A 90kDa and 4-HNE 66kDa). A series of negative control sections were incubated in primary antibody alone, secondary antibody alone, or buffers only. Sections were washed (2 × 5 min each in buffer 2 then PBS), mounted on gelatinized slides and air-dried. Slides were rinsed with PBS, counterstained with DAPI nuclear stain (Sigma-Aldrich, 1:2000 in dH_2_O; 30 s) rinsed twice with dH_2_O and cover slipped with DABCO fluorescent mounting medium (Sigma-Aldrich; 0.2% in 90% glycerol in PBS) then stored at 4 °C.

**FIGURE 1:**
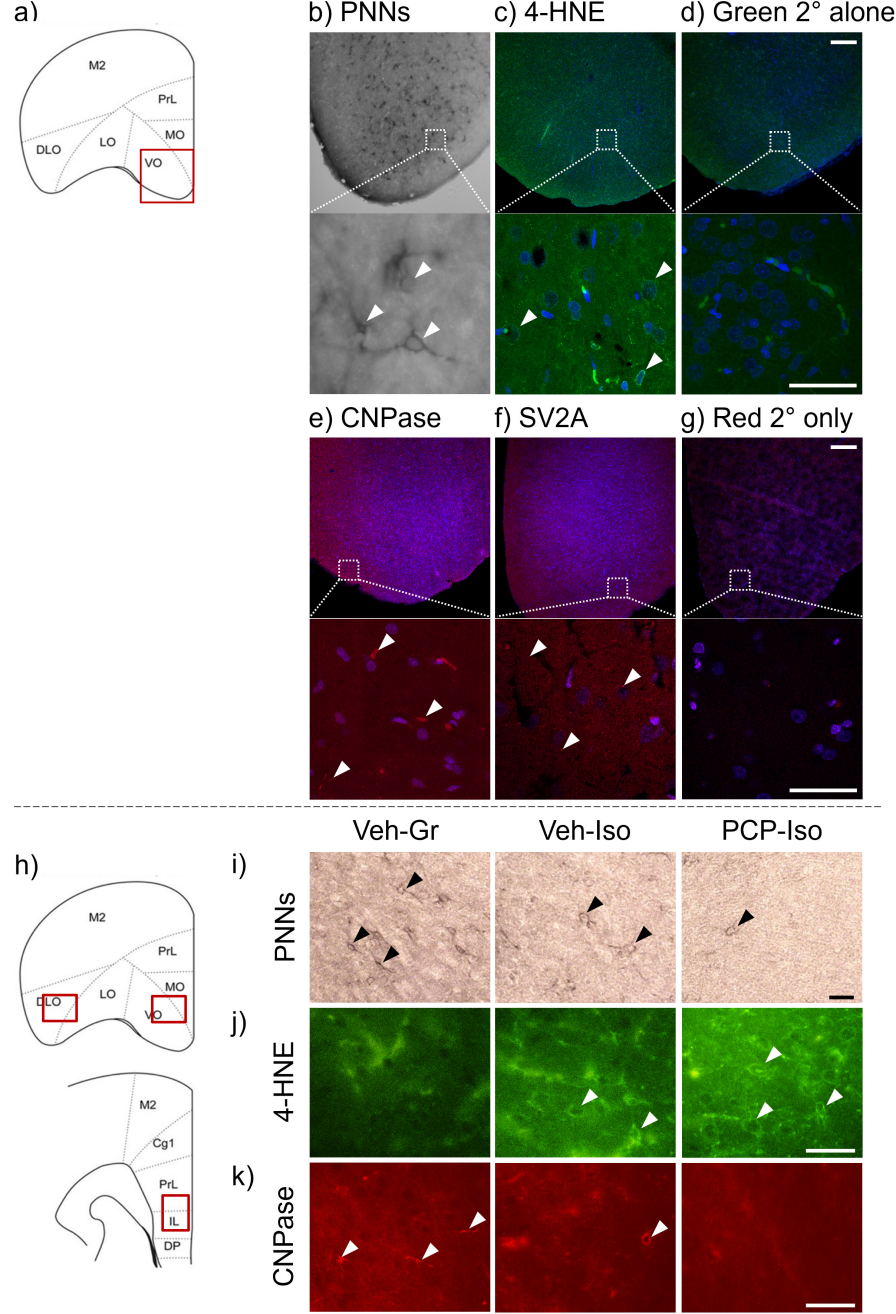
Lectin-binding and immunohistochemical staining for perineuronal nets (PNNs) and markers of lipid peroxidation (4-HNE), oligodendrocytes and myelination (CNPase) and synaptic vesicle density (SV2A). To provide qualitative insight into the morphology of labeled features confocal microscopy images were obtained **(a)** at low (x10; solid red border) and high [x63; white dashed borders in panel **(b–g)**] magnifications from the medial/ventral orbitofrontal cortex (MO/VO) after incubation with **(b,c,e,f)** primary and secondary or **(d,g)** secondary antibodies alone. Images are maximum intensity projections. For quantitative analysis standard fluorescence microscopy images were collected from **(h)** consistently locations within the MO/VO, lateral/dorsolateral orbitofrontal (LO/DLO) and prelimbic/infralimbic (PrL/IL) regions of the frontal cortex (Paxinos and Watson, 2006). To aid placement of these images within the figure only one quarter of their size is presented. For PNNs and 4-HNE manual counts of labeled structures [three examples marked by arrowheads in **(b,c)**] were captured using a cell counter plugin, while for CNPase and SV2A automated detection settings were used enable the density or area of immunoreactive features [three examples marked by arrowheads in panel **(e,f)**] to be quantified. Representative images from the MO/VO show **(i)** fewer PNNs, **(j)** more 4-HNE-positive cells, and **(k)** a lower area of CNPase immunoreactivity in rats that received PCP on postnatal day (PND) 7, 9, and 11 and were housed in isolation from weaning on PND 21 (PCP-Iso), compared to rats that received vehicle injections and were housed in groups (Veh-Gr) or isolation (Veh-Iso). Scale bars are 200 μm in low magnification confocal images **(b–g**, upper panels) and 50 μm for the remainder. CNPase, 2′,3′-cyclic nucleotide 3′-phosphodiesterase; 4-HNE, 4-hydroxynonenal; PNN, perineuronal net; SV2A, synaptic vesicle glycoprotein 2A.

To provide qualitative insights into the morphology of labeled features within the medial/ventral orbitofrontal cortex (MO/VO; Figure 1a) a small number of sections were viewed using a Zeiss LSM 880 confocal microscope with 405, 488, and 561 nm lasers for blue, green and red channels, respectively. The 488 nm laser was also used to obtain brightfield-like images. Representative × 10 (Figures 1b–g; upper panels) and x63 images (Figures 1b–g; lower panels) were collected with 0.3NA and 1.4NA objectives, using Zen 2.1 SP3 software (Zeiss). For quantitative analysis sections were viewed on a Nikon EFD-3 microscope equipped with brightfield and fluorescence options. Consistently placed snapshots (× 10 for PNNs, × 20 for all other markers) were obtained from the MO/VO, lateral/dorsolateral orbitofrontal (LO/DLO) and prelimbic/infralimbic (PrL/IL) cortices (Figure 1h) of all rats using a Spot Insight 5MP CM05 USB camera and Spot Advanced software (v5.6; Diagnostic Instruments Inc.). Anatomical boundaries were determined using the stereotaxic brain atlas of Paxinos and Watson (2006). The number of PNNs per DAB-stained image (Figure 1i) were manually captured via a cell Counter plugin for ImageJ 1.54^1^ (Reichelt et al., 2021). The mean maximal distance of PNN extension from the soma and mean maximal PNN thickness at soma level (Smith et al., 2015) were measured for the three most extensive PNNs per image, using the Analyze > Measure tool in ImageJ 1.54 (after setting an appropriate scale; 3.5408 pixels/μm). The relatively small numbers of 4-HNE-positive cells per fluorescent image (Figure 1j) were manually counted as described above, and larger numbers of SV2A-postiive puncta per image were automatically counted with Fiji (Windows 32-bit; Schindelin et al., 2012) by customizing an established protocol (Labno, 2014) to reflect optimal detection settings for this marker. The area of CNPase immunoreactivity (Wang et al., 2007; Figure 1k) was quantified by a similar threshold-based approach after setting the scale (7.0816 pixels/μm). Because it is theoretically possible there might be a change in the extent of immunoreactivity within labeled features without any change in the number or area of features per image, the intensity of immunoreactivity in fluorescent images was also determined automatically, using the Analyze > Color Histogram tool in Fiji and normalized by subtraction of background staining (Goh et al., 2020). To provide reassurance that changes to each marker of interest were not a simple consequence of global alterations in cell density the number of DAPI-labeled nuclei per image was automatically counted with Fiji, as described above for SV2A. Data for each rat and brain region were averaged across the four sections per marker, such that n represents the number of biological and not technical replicates.

### 2.3 Statistical analysis

No exclusion criteria were set and data from all animals were included in the analyses. These were planned before the study took place (although not formally registered) and performed using GraphPad Prism v10.3.0. Normality was assessed with D’Agostino-Pearson or Kolmogorov-Smirnov tests. Based on the outcomes, PNN data, nuclei counts and the intensity of 4-HNE, CNPase and SV2A immunoreactivity were analyzed by mixed-effects model (with neurodevelopmental condition as a between-subjects factor and frontal cortical sub-region as a within-subjects factor). The Geisser-Greenhouse correction for unequal variance was applied and ANOVAs were followed with Tukey’s multiple comparisons *post-hoc* test. 4-HNE and SV2A counts as well as CNPase area data were analyzed by non-parametric Kruskal-Wallis tests (applied separately to each frontal cortical sub-region, with neurodevelopmental condition as the sole factor) followed by Dunn’s *post hoc*. Data analyzed with parametric tests are presented as mean ± standard error of the mean (SEM) and those analyzed with non-parametric tests as median ± interquartile range (IQR). *P* < 0.05 was considered statistically significant.

## 3 Results

### 3.1 Validation of lectin-binding and immunohistochemical staining

The selected lectin and primary antibodies have been used to visualize PNN (Seeger et al., 2004), 4-HNE (Fragoulis et al., 2017), CNPase (Fan et al., 2020) and SV2A (Xiao et al., 2024) staining in rodent brain, and patterns in this study are consistent with previous observations in the rat frontal cortex (Seeger et al., 2004; Tan et al., 2012; Luo et al., 2019; Halff et al., 2021). Thus, PNN labeling (Seeger et al., 2004) surrounded somas, proximal dendrites and initial axon segments (Figures 1b,i) and enabled counting of enwrapped cells as well as morphological analyses of PNN integrity. 4-HNE immunoreactivity (Tan et al., 2012) occurred within somas but was absent from nuclei (Figures 1c,j). Although labeling was relatively faint with the antibody concentrations used here it was still sufficient to enable manual cell counting. CNPase immunoreactivity (Figures 1e,k) was consistent with reported labeling of the cytoplasm and plasma membrane (Trapp et al., 1988) and was evident in somas and processes (albeit at lower levels than following sodium citrate antigen retrieval, 40 h longer incubation in primary antibody, and higher temperature incubation in secondary antibody with amplification via avidin-biotin complexation; Luo et al., 2019). The presence of immunoreactive processes prevented cell counting, since it was not feasible to distinguish whether each of these were from the same or different cells, however the area of immunoreactivity could be readily determined. SV2A immunoreactivity (Figure 1f) formed a punctate pattern that was most abundant in perisomatic regions but absent from within soma or nuclei (Halff et al., 2021) and could be automatically counted. Confidence in labeling of each marker is maximized by that fact that it only occurred in sections incubated with lectin or primary antibody plus the respective label, and was abolished by the absence of primary antibodies (Figures 1d,g) or secondary antibodies (data not shown).

### 3.2 No impact of combined neonatal PCP and isolation rearing on total cell density

There was no impact of neurodevelopmental condition on the density of DAPI-stained nuclei [F(2,33) = 1.394, *P* = 0.2624], no neurodevelopmental condition × frontal cortical sub-region interaction [F(4,66) = 1.394, *P* = 0.7618], nor any differences between individual groups. Mean ± SEM densities for Veh-Gr, Veh-Iso, and PCP-Iso, respectively were 213 ± 5, 211 ± 4 and 208 ± 4 in the MO/VO, 216 ± 4, 211 ± 3 and 204 ± 3 in the LO/DLO, and 196 ± 5, 199 ± 3 and 194 ± 3 in the PrL/IL.

### 3.3 Impact of combined neonatal PCP and isolation rearing on PNNs

**FIGURE 2:**
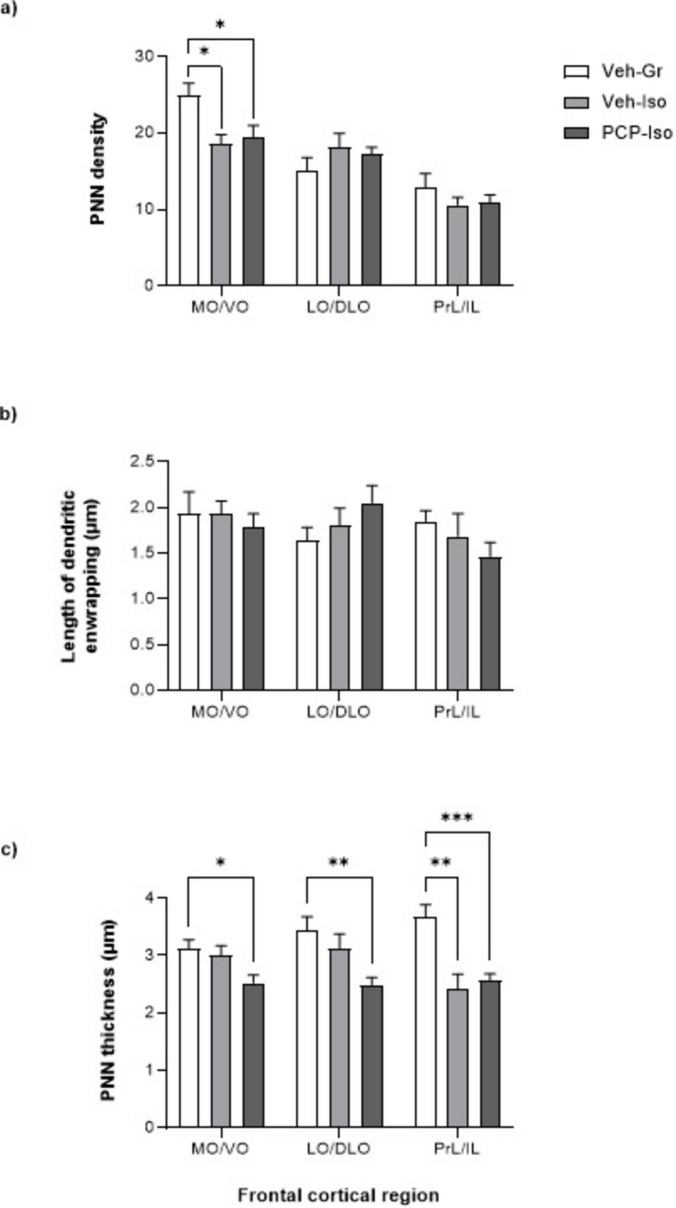
Impact of combined neonatal phencyclidine (PCP) and isolation rearing on perineuronal nets (PNNs) in the frontal cortex. Mean ± SEM **(a)** density of PNNs **(b)** length of the longest three dendritic enwrapments per image and **(c)** depth of the thickest three soma enwrapments per image. Male Lister hooded rats received saline vehicle (1 mL/kg s.c.; Veh) or PCP (10 mg/kg) on postnatal day (PND) 7, 9, and 11 were housed in groups (Gr) or isolation (Iso) from weaning on PND 21. They underwent novel object discrimination (NOD) three separate times at 1–2 weeks intervals (PND 57–80) following acute i.p. administration of 0.5% methylcellulose 1% Tween-80 vehicle (1 mL/kg), SB-399885 (10 mg/kg) or MMPIP (10 mg/kg) on separate days using a cross-over design. Tissue was collected on PND 79–80 (*n* = 11–13 per neurodevelopmental condition) and lectin binding data were obtained from consistently placed regions of interest within medial/ventral orbitofrontal (MO/VO), lateral/dorsolateral orbitofrontal (LO/DLO) and prelimbic/infralimbic (PrL/IL) sub-regions. *P < 0.05; **P < 0.01; ***P < 0.001 Veh-Iso or PCP-Iso versus Veh-Gr (mixed-effects model with Geisser-Greenhouse corrections for unequal variance, followed by Tukey’s *post hoc*). PNN, perineuronal net.

There was a neurodevelopmental condition x frontal cortical sub-region interaction for PNN density [F(4,54) = 3.598, *P* = 0.0113], which was reduced in the MO/VO of both single-hit Veh-Iso and dual-hit PCP-Iso (−25 and −22%; *P* < 0.05 versus Veh-Gr control, Figure 2a). Although the length of dendritic enwrapping was not influenced by neurodevelopmental condition (Figure 2b), the thickness of soma enwrapment did show a main effect of neurodevelopmental condition [F(2,32) = 13.03, *P* < 0.0001] plus a neurodevelopmental condition x frontal cortical sub-region interaction [F(4,60) = 3.626, *P* = 0.0103]. The thickness of soma enwrapment was lower in the PrL/IL of both single-hit Veh-Iso (−35%; *P* < 0.01 versus Veh-Gr control) and dual-hit PCP-Iso (−30%; *P* < 0.001 versus Veh-Gr control; Figure 2c). Of note, PCP-Iso showed additional decreases in both the MO/VO (−19%; *P* < 0.05) and LO/DLO (−28%; *P* < 0.01), where Veh-Iso showed no change (Figures 1i, 2c).

### 3.4 Impact of combined neonatal PCP and isolation rearing on 4-HNE immunoreactivity

**FIGURE 3:**
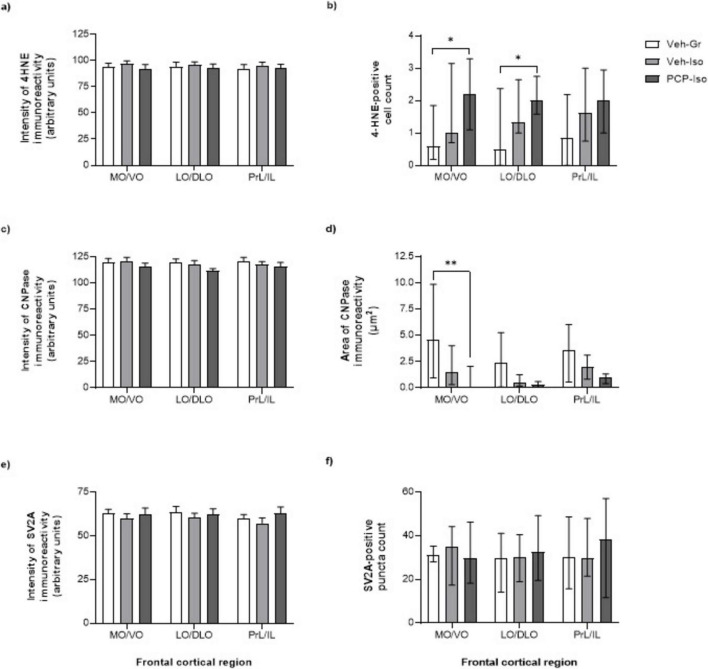
Impact of combined neonatal phencyclidine (PCP) and isolation rearing on markers of lipid peroxidation (4-HNE), oligodendrocytes and myelination (CNPase) and synaptic vesicle density (SV2A) in the frontal cortex. Mean ± SEM intensity of **(a)** 4-HNE, **(c)** CNPase and **(e)** SV2A immunoreactivity, together with median ± IQR **(b)** density of 4-HNE-positive cells, **(d)** area of CNPase immunoreactivity and **(f)** density of SV2A-labeled puncta. Male Lister hooded rats received saline vehicle (1mL/kg s.c.; Veh) or PCP (10 mg/kg) on postnatal day (PND) 7, 9, and 11 were housed in groups (Gr) or isolation (Iso) from weaning on PND 21. They underwent novel object discrimination (NOD) three separate times at 1–2 week intervals (PND 57-80) following acute i.p. administration of 0.5% methylcellulose 1% Tween-80 vehicle (1 mL/kg), SB-399885 (10 mg/kg) or MMPIP (10 mg/kg) on separate days using a cross-over design. Tissue was collected on PND 79-80 (*n* = 11–13 per neurodevelopmental condition) and immunohistochemical data were obtained from consistently placed regions of interest within medial/ventral orbitofrontal (MO/VO), lateral/dorsolateral orbitofrontal (LO/DLO) and prelimbic/infralimbic (PrL/IL) sub-regions. *P < 0.05; **P < 0.01 PCP-Iso versus Veh-Gr (**a**, **c**, **e** mixed-effects model with Geisser-Greenhouse corrections for unequal variance followed by Tukey’s *post hoc*, or **b**, **d**, **f** Kruskal-Wallis test with Dunn’s *post hoc*). CNPase, 2′,3′-cyclic nucleotide 3′-phosphodiesterase; 4-HNE, 4-hydroxynonenal; SV2A, synaptic vesicle glycoprotein 2A.

There was no effect of neurodevelopmental condition on the intensity of 4-HNE immunoreactivity (Figure 3a). However, there was a main effect of neurodevelopmental condition on the density of 4-HNE positive cells in the MO/VO (Kruskal-Wallis statistic = 6.645, *P* = 0.0361) and LO/DLO (Kruskal-Wallis statistic = 6.040, *P* = 0.0488). In both these sub-regions 4-HNE counts were elevated in PCP-Iso (3.8- to 4-fold; *P* < 0.05 versus Veh-Gr control) but not Veh-Iso (Figures 1j, 3b).

### 3.5 Impact of combined neonatal PCP and isolation rearing on CNPase immunoreactivity

There was no effect of neurodevelopmental condition on the intensity of CNPase immunoreactivity (Figure 3c). Nevertheless, there was a main effect of neurodevelopmental condition on the area of labeling in the MO/VO (Kruskal-Wallis statistic = 10.77, *P* = 0.0046). This was reduced (−85%; *P* < 0.01) in PCP-Iso but unaltered in Veh-Iso (Figures 1k,3d).

### 3.6 Impact of combined neonatal PCP and isolation rearing on SV2A immunoreactivity

There was no effect of neurodevelopmental condition on the intensity of SV2A immunoreactivity (Figure 3e) or the density of labeled puncta (Figure 3f).

### 3.7 Summary of frontal cortical changes following combined neonatal PCP and isolation rearing

The sections analyzed here came from the same animals as used for our previous assessments of GABAergic and inflammatory markers (Cale et al., 2024), and findings for the cohort as a whole are summarized in Table 1. Single-hit Veh-Iso showed reduced parvalbumin expression and reduced WFA labeling of PNNs, predominantly in the PrL/IL. These were more extensive in dual-hit PCP-Iso, where they extended to the orbitofrontal cortex and were accompanied by elevations in microglial activation, cytokine levels, and lipid peroxidation, as well as reduced expression of the mature oligodendrocyte and myelination marker, CNPase.

**TABLE 1:** Summary of frontal cortical changes following combined neonatal phencyclidine (PCP) and isolation rearing.

Marker	Veh-Iso		PCP-Iso	
	OFC	PrL/IL	OFC	PrL/IL
Parvalbumin	–	↓ Intensity and count	↓ Intensity	↓ Intensity and count
Calbindin	–	–	–	–
Somatostatin	–	–	–	–
PNNs	↓ Count	↓ Thickness	↓ Count and thickness	↓ Thickness
Iba-1	–	–	↑ Rod morphology	–
IL-6	-		↑ Level (whole homogenate)	
4-HNE	–	–	↑ Count	–
CNPase	–	–	↓ Area	–
SV2A	–	–	–	–

↑, increase; ↓, decrease; –, no change. CNPase, 2′,3′-cyclic nucleotide 3′-phosphodiesterase; 4-HNE, 4-hydroxynonenal; Iba-1, ionized calcium binding adapter molecule 1; OFC, orbitofrontal cortex; PNN, perineuronal net; PrL/IL, prelimbic/infralimbic cortex; SV2A, synaptic vesicle glycoprotein 2A. For full details of parvalbumin, calbindin, somatostatin, Iba-1 and IL-6 measures see Cale et al. (2024).

## 4 Discussion

Improved preclinical models are essential to enable more predictive evaluation of novel therapeutics targeting negative valence, cognitive and social components of the RDoC. The dual-hit combination of neonatal PCP followed by isolation rearing has already been reported to produce a wider range of behavioral and neurochemical changes than single-hit isolation rearing, and evidence to date suggests that the alterations in PCP-Iso are more akin to those in schizophrenia (Gaskin et al., 2011, 2014; Kohli, 2018; Shortall et al., 2020; Cale et al., 2024). The current histochemical study is the first to show the combined presence of PNN deficits, increased lipid peroxidation and reductions in mature oligodendrocytes and/or myelination within the frontal cortex of PCP-Iso. These findings further support relevance of the dual-hit PCP-Iso model.

Some of the individual markers measured here have been examined in related neurodevelopmental models. For example, a dual-hit approach consisting of maternal separation (4 h/day from PND 2-20) followed by transient social isolation (PND 21-35) failed to alter adulthood PNN densities in the frontal cortex of male Sprague-Dawley rats (Gildawie et al., 2021). Similarly, prolonged social isolation of male CrL:CD1 mice (PND 21-109) also failed to impact on frontal cortical PNN densities (Biro et al., 2023) when compared to a somewhat unusual group of control animals that underwent single housing throughout adulthood behavioral testing (PND 64-109). In a dual-hit design more like that used herein male FVB mice from the Veh-Iso group (PND 21-90) did show reduced PNN counts in the retrosplenial cortex (Klimczak et al., 2021), despite male mice generally being less gregarious than rats (Lidster et al., 2019) so potentially less impacted by isolation. In the same study the combination of neonatal MK-801 (PND 7) plus isolation also reduced PNN counts (Klimczak et al., 2021) and triggered excitatory-inhibitory imbalance (Castillo-Gómez et al., 2017), but to the best of our knowledge the inflammatory changes reported in PCP-Iso (Cale et al., 2024) or alterations in 4-HNE and CNPase are yet to be examined there. There are similar gaps in knowledge for maternal separation plus transient social isolation in Sprague-Dawley rats, and for maternal exposure of Wistar Han rats to a high fat diet. The former elevated 4-HNE expression in the hippocampus although frontal cortical changes have yet to be examined (Chen et al., 2025), and the latter reduced frontal cortical CNPase expression in male offspring during adolescence and adulthood (Frankowska et al., 2024). More complete histochemical data are available for low protein-induced intrauterine growth restriction in Sprague-Dawley rats, which reduced parvalbumin and PNN markers, and increased microglial activation and oxidative stress in the anterior cingulate cortex (ACC) of adolescent male offspring (PND 35; Allgäuer et al., 2023). This model therefore has much in common with PCP-Iso, but its predictive validity is less well-established because associated behavioral changes have not undergone the same level of pharmacological investigation with antipsychotics (Watson et al., 2016; Hamieh et al., 2021), the voltage-gated sodium channel blocker lamotrigine (Gaskin et al., 2016), glycine transporter 1 inhibitor RO4993850 (Kohli, 2018), and 5-HT_6_ receptor antagonist SB-399885 (Shortall et al., 2020). Few studies have examined preclinical SV2A expression but interestingly it is upregulated in the frontal cortex of male Wistar rats resilient to the effects of adulthood social isolation (PND ∼75-135) on sucrose preferences, and unaltered in those susceptible to the effects of isolation (Filipović et al., 2024).

Perineuronal net reductions and altered expression of 4-HNE and CNPase in the frontal cortex of PCP-Iso mimic several post-mortem findings from patients with psychosis or schizophrenia over the last 22 years. Three studies examined PNNs in frontal cortical regions and all found reductions. These range from −25% in the dorsolateral prefrontal cortex as a whole (Alcaide et al., 2019) to −48% in Brodmann’s area (BA) 9 (Enwright et al., 2016) of predominantly middle-aged individuals with mean ± SEM illness durations of 22.1 ± 5 years. Even larger reductions of −70% and −76% were noted in BA9 layers 3 and 5 when old age patients were included (Mauney et al., 2013). Only two studies examined 4-HNE expression, and both found elevations (30%–50%) in BA10 (Andreazza et al., 2013) or the ACC (Wang et al., 2009) of middle to old age patients. Interestingly there is a suggestion of more extensive 4-HNE elevations (72%) as well as more extensive PNN decreases in medication-free individuals (Wang et al., 2009; Mauney et al., 2013) although numbers for these subgroup analyses are low. Five studies examined CNPase expression, also in middle to old age, and decreases of around 30% were observed for BA10 as a whole (Wesseling et al., 2014) as well as BA10 gray matter (Flynn et al., 2003) and BA9 white matter (Hof et al., 2003) when analyzed via ELISA, mass spectrometry or immunohistochemistry. The two studies where decreases failed to reach significance (∼17%) both used western blotting (Mitkus et al., 2008; Hercher et al., 2014), and one acknowledged a potential confound arising from high CNPase expression in subgroups who disclosed moderate to heavy alcohol or other substance use (Hercher et al., 2014).

Positron emission tomography (PET) ligands for SV2A have provided new insights into presynaptic vesicle changes in schizophrenia over the last 5 years. In chronic cases (mean ± SEM ages 41.5 ± 2.7, 40.9 ± 2.8, or 40.5 ± 3.1 years, scanned 17.3 ± 3.4 years after onset of illness) there are consistent widespread reductions in [^11^C]UCB-J volume of distribution (VT). This measure reflects both specific and non-specific binding, and is decreased by 8%–13% in the frontal cortex and/or ACC (Cohen’s d effect sizes 0.8–0.9; Onwordi et al., 2020, 2021; Radhakrishnan et al., 2021). In contrast first-episode cases (aged 26.5 ± 1.7 and scanned 2.7 ± 0.5 years after onset) show no change in VT (Onwordi et al., 2024), and alterations to other measures are less consistent. Distribution volume ratio (DVR) adjusts for non-specific binding using a reference region approach so is less variable than VT (Onwordi et al., 2021). Although decreases of 11% have been detected in the ACC (Onwordi et al., 2025) there are also negative reports in the dorsolateral prefrontal cortex (Onwordi et al., 2024). Binding potential (BPND) reflects non-displaceable specific binding and appears more sensitive, being decreased by 18%–19% in the orbitofrontal cortex and right middle frontal gyrus (Cohen’s d 1.5; Yoon et al., 2023). One recent study using the alternative tracer [^18^F]SynVesT-1 even showed reduced BPND in the ACC and prefrontal cortex at an earlier stage in first-episode psychosis (26.1 ± 1.2 years, equivalent to 0.9 ± 0.2 years after onset), plus the ACC of those at clinical high risk (21.2 ± 0.8 years), albeit with smaller effect sizes (Cohen’s d 0.29–0.35) and an acknowledgment that heterogeneity of early psychosis and limited diagnostic stability may affect generalizability (Blasco et al., 2025). It is unclear which PET measures might align most closely with immunolabeling of SV2A. However, the closest rodent equivalent of the dorsolateral prefrontal cortex is the PrL/IL (Uylings et al., 2003), and since rats were young adults at the time of tissue collection the lack of SV2A change here may parallel clinical reports of unchanged [^11^C]UCB-J DVR in this region (Onwordi et al., 2024). Declarative memory deficits occur before the clinical onset of psychosis (Seidman et al., 2016) and although they are well-established in PCP-Iso by PND 60 (Shortall et al., 2020) other components of the syndrome may not emerge until PND 72 (Gaskin et al., 2014). These include sensorimotor deficits detected by assessing pre-pulse inhibition of the acoustic startle response. Because one human year equates to 11.8 days in an adult rat (Sengupta, 2013) the PCP-Iso timepoint corresponding to the majority of first-episode SV2A PET scans (Yoon et al., 2023; Onwordi et al., 2024, 2025) is actually PND 104-112 rather than PND 79–80. Future studies should therefore assess additional components of the RDoC (e.g., additional cognitive domains, social processes and sensorimotor gating) as well as SV2A expression at this later stage, and should be expanded to include the ACC. Unfortunately few sections available for the current study included this region and although a preliminary analysis (typically 1-2 sections per rat) suggested potential emergence of low SV2A subgroup in the ACC of PCP-Iso (Esuaikoh et al., 2024) the data are not presented here because we did not have sufficient sections for equivalent assessments of 4-HNE or CNPase.

**FIGURE 4:**
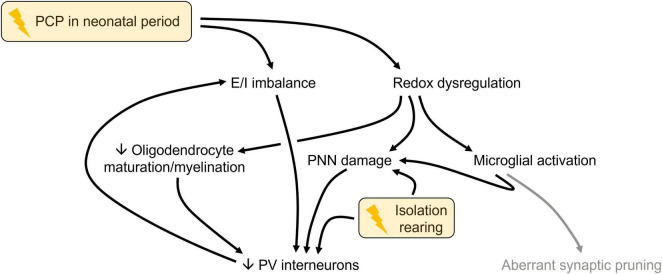
Proposed framework linking the frontal cortical impacts of combined neonatal phencyclidine (PCP) and isolation rearing. Responses to each intervention (yellow boxes) that are evident by PND 79–80 are shown in black. Aberrant synaptic pruning (gray) is a consequence of earlier alterations so it is plausible that decreased SV2A expression may emerge later, despite not being evident on PND 79–80. E/I, excitatory/inhibitory; PND, postnatal day; PNN, perineuronal net; PV, parvalbumin.

The current master hypothesis for schizophrenia (Howes and Onwordi, 2023) proposes that multiple early-life events trigger aberrant microglial-mediated synaptic pruning, which leads to cortical excitation-inhibition imbalance (underlying the negative and cognitive symptoms) plus disinhibition of striatal and midbrain dopaminergic neurons (causing psychotic symptoms, the stress of which further exacerbates aberrant synaptic pruning). In this scenario, increased microglial activation would precede reduced SV2A expression. Efforts to test this prediction via imaging of ultra-high risk, first-episode and chronic illness populations have been hampered by availability of suitable PET tracers. For example, there are no labels for HLA-DR or Iba-1 (which are elevated at post-mortem; Radewicz et al., 2000; Wierzba-Bobrowicz et al., 2005; Fillman et al., 2013; Hercher et al., 2014) and in multiple studies those for 18 kDa translocator protein (TSPO; e.g., [^11^C](R)-PK11195) failed to show changes in any frontal cortical subregion in any stage of illness (Holmes et al., 2016; Di Biase et al., 2017; Conen et al., 2021). This is potentially because TSPO expression (which is not specific to microglia) is actually not upregulated in post-mortem samples from patients with schizophrenia or in activated microglial cultures (Sneeboer et al., 2020). However, preclinical models like PCP-Iso are able to provide temporal insight. Current findings support a framework (Figure 4) based on the role of redox dysregulation in schizophrenia (Perkins et al., 2020), whereby NMDA receptor hypofunction during development triggers excitatory/inhibitory imbalance and loss of antioxidant control. Oxidative stress in turn leads to microglial activation, PNN degradation and impaired oligodendrocyte differentiation and myelin maturation. These converge to impact on parvalbumin-positive interneurons (which along with PNNs are also affected by isolation as a single intervention), and their loss further exacerbates excitatory/inhibitory imbalance and perpetuates the negative cycle (Perkins et al., 2020).

In light of current support for the face validity of PCP-Iso we now propose some additional characterization studies to further enhance its utility and generalizability. The main limitation of the model is that so far only males have been examined. There are recognized sex differences in responses to neonatal administration of NMDA receptor antagonists (e.g., Zhao et al., 2013) and isolation rearing (e.g., Fabricius et al., 2010; Logue et al., 2022) as single interventions, so it is probable they would also occur following the dual-hit combination. There are also baseline behavioral differences between normal male and female rats, for example during NOD testing of declarative memory group housed Lister hooded females appear to retain information for longer (McLean et al., 2010) than males (King et al., 2004). Careful modification of task protocols would therefore be required to ensure equivalent sensitivity to cognitive deficits in both sexes of PCP-Iso. Separate characterization studies should assess brain regional volumetric changes, particularly those affecting the frontal cortical locations focused on here. Patients with schizophrenia exhibit both cortical and subcortical alterations (e.g., Kirschner et al., 2022), and structural MRI (Schubert et al., 2009) or stereological approaches (Fabricius et al., 2010) confirm that some of these also occur in rats following isolation rearing as a single intervention. Longitudinal MRI examinations in PCP-Iso would provide valuable new insights into the developmental progression of such changes. Incidentally in its current form our so-called “dual-hit” model may more accurately reflect a “triple-hit” combination, whereby movement of pups from the breeder to a new facility on PND 3 represents an initial developmental manipulation during the sensitive perinatal period (due to transport stress in the pups themselves and/or disruption of maternal care in transported dams). The same transport exposure also occurred in our Veh-Gr and Veh-Iso so does not confound our current findings, but we acknowledge that the Veh-Gr controls actually experienced a “single-hit” perinatal stressor. This could not be mitigated by purchasing pregnant females instead of lactating dams with pups, as this would simply transfer any impact to another sensitive developmental window (Ratajczak et al., 2015; Abramova et al., 2024). Requesting commercial breeders perform neonatal dosing and isolation rearing then transporting animals after sensitive neurodevelopmental windows have closed would limit scope for the longitudinal examinations proposed above, so the only possible alternative would be to establish an in-house breeding colony to allow complete elimination of transport stress. Other researchers wishing to employ this procedural variation should be mindful that the resulting phenotype has yet to be characterized and may not be as robust as that observed in 100% of our 10 PCP-Iso cohorts to date. In addition to recommending use of PCP-Iso for future examinations of novel therapeutics we also advocate including the current histochemical endpoints alongside behavioral ones. The PCP-Iso model should be sensitive to agents designed to support parvalbumin-positive neurons and PNNs, promote myelination, or normalize redox dysregulation. One priority should be positive modulators of Kv3.1/3.2 voltage-gated potassium channels (e.g., AUT00206), which normalize parvalbumin-positive cell counts in alternative models for schizophrenia (Leger et al., 2015) and have recently shown promising effects on gamma oscillations in patients (Kaar et al., 2024). Other suggestions include matrix metalloproteinase 2/9 (MMP2/9) inhibitors (e.g., SB-3CT) which overcome microglial, parvalbumin and PNN changes in a transgenic model of redox dysregulation when administered during adolescence (Dwir et al., 2020). Likewise the sphingosine-1 phosphate receptor antagonist fingolimod increased the density of parvalbumin-positive neurons and had subtle effects on WFA-positive PNNs in normal adult mice (Ueno et al., 2022), reduced microglial activation in a cuprizone demyelination model (Li et al., 2023) and had promising effects on negative symptoms in a small clinical trial (Karbalaee et al., 2023). Meta-analyses of adjunctive treatment with *N*-acetylcysteine (a precursor of the endogenous antioxidant glutathione) have shown mixed results (e.g., Zhang et al., 2024; Kishi et al., 2023) but there is interest in alternative approaches including nicotinamide adenine dinucleotide precursors that target antioxidant and inflammatory pathways as well as mitochondrial function (Qader et al., 2025). Another interesting line of research, if deficits in SV2A expression occur after PND 80, should involve the antiepileptic agents levetiracetam and brivaracetam. These compounds interact with SV2A (Danish et al., 2017), appear to normalize the consequence of increases or decreases in its expression during studies of neuronal excitability (Czapińska-Ciepiela et al., 2024), and levetiracetam had encouraging effects across all schizophrenia symptoms domains in a small clinical trial (Behdani et al., 2022). In conclusion, the combined presence of GABAergic and inflammatory changes (Cale et al., 2024), increased lipid peroxidation, plus reduced perineuronal net and oligodendrocyte markers in the frontal cortex of PCP-Iso enhance the face validity of this neurodevelopmental model for schizophrenia, and support its use to further elucidate disease neurobiology and select plausible new targets for drug development.

## Data Availability

The raw data supporting the conclusions of this article will be made available by the authors, without undue reservation.

## Footnotes

1
https://imagej.net/plugins/cell-counter
